# Clinical and molecular characterization of three patients with Hepatocerebral form of mitochondrial DNA depletion syndrome: a case series

**DOI:** 10.1186/s12881-019-0893-9

**Published:** 2019-10-29

**Authors:** Ghazale Mahjoub, Parham Habibzadeh, Hassan Dastsooz, Malihe Mirzaei, Arghavan Kavosi, Laila Jamali, Haniyeh Javanmardi, Pegah Katibeh, Mohammad Ali Faghihi, Seyed Alireza Dastgheib

**Affiliations:** 1 Persian BayanGene Research and Training Center, Shiraz University of Medical Sciences, Shiraz, Iran; 2 Student Research Committee, Shiraz University of Medical Sciences, Shiraz, Iran; 3 Italian Institute for Genomic Medicine (IIGM), University of Turin, Turin, Italy; 4 Department of Pediatrics, Shiraz University of Medical Sciences, Shiraz, Iran; 5 Center for Therapeutic Innovation, Department of Psychiatry and Behavioral Sciences, University of Miami Miller School of Medicine, Miami, USA; 6 Department of Medical Genetics, School of Medicine, Shiraz University of Medical Sciences, Shiraz, Iran

**Keywords:** Mitochondrial DNA depletion syndrome, *DGUOK*, *MPV17*, Mitochondrial disorders

## Abstract

**Background:**

Mitochondrial DNA depletion syndromes (MDS) are clinically and phenotypically heterogeneous disorders resulting from nuclear gene mutations. The affected individuals represent a notable reduction in mitochondrial DNA (mtDNA) content, which leads to malfunction of the components of the respiratory chain. MDS is classified according to the type of affected tissue; the most common type is hepatocerebral form, which is attributed to mutations in nuclear genes such as *DGUOK* and *MPV17*. These two genes encode mitochondrial proteins and play major roles in mtDNA synthesis.

**Case presentation:**

In this investigation patients in three families affected by hepatocerebral form of MDS who were initially diagnosed with tyrosinemia underwent full clinical evaluation. Furthermore, the causative mutations were identified using next generation sequencing and were subsequently validated using sanger sequencing. The effect of the mutations on the gene expression was also studied using real-time PCR. A pathogenic heterozygous frameshift deletion mutation in *DGUOK* gene was identified in parents of two affected patients (c.706–707 + 2 del: p.k236 fs) presenting with jaundice, impaired fetal growth, low-birth weight, and failure to thrive who died at the age of 3 and 6 months in family I. Moreover, a novel splice site mutation in *MPV17* gene (c.461 + 1G > C) was identified in a patient with jaundice, muscle weakness, and failure to thrive who died due to hepatic failure at the age of 4 months. A 5-month-old infant presenting with jaundice, dark urine, poor sucking, and feeding problems was also identified to have another novel mutation in *MPV17* gene leading to stop gain mutation (c.277C > T: p.(Gln93*)).

**Conclusions:**

These patients had overlapping clinical features with tyrosinemia. MDS should be considered a differential diagnosis in patients presenting with signs and symptoms of tyrosinemia.

## Background

Mitochondrial diseases are clinically and phenotypically heterogeneous disorders caused by defects in mitochondrial DNA (mtDNA) or nuclear genes encoding proteins directly or indirectly involved in mtDNA maintenance (respiratory subunits, assembly factors, enzymes, etc.). A wide range of mutations has so far been reported in patients affected with mitochondrial disorders [1–5].

MDS is inherited as autosomal recessive disorder and mainly has an early onset. It is associated with a notable reduction in mtDNA content, which causes inappropriate function of the respiratory chain components affecting a specific tissue or multiple organs such as muscle, liver, brain, and kidney [6, 7]. Regarding the affected organs, MDS is classified into four categories: myopathic, encephalomyopatic, hepatocerebral, and neurogastrointestinal forms. Each type results from different nuclear genes mutations (Additional file 1: Table S1). It has been reported that these genes have major roles in nucleotide synthesis and replication of mtDNA and their mutations disrupt mtDNA maintenance [8, 9].

Hepatocerebral type of MDS is the most common form caused by mutations in the following nuclear genes: *TWNK*, *POLG*, *DGUOK*, *MPV17,* and *TFAM* [2, 3, 10]. The hepatocerebral type generally occurs in infants of less than 6 months of age and usually results in death within the first year of life, chiefly due to hepatic failure [11].

Mutations in *DGUOK*, encoding the mitochondrial deoxyguanosine kinase, causes MDS type 3, an early-onset disease belonging to hepatocerebral form. This enzyme phosphorylates purine nucleotides to nucleotide monophosphates and provides balanced supply of nucleotides necessary for mtDNA replication [12]. *DGUOK* is a ubiquitously expressed gene with the highest expression in muscle, brain, liver, and lymphoid tissues [13].

The affected neonates are mostly diagnosed with hepatic and neurological defects, lactic acidosis, and hypoglycemia in the first few weeks of life. They present with progressive liver disease (the most common cause of death), low-birth weight, and neurological impairments (e.g., myopathy, developmental delay, nystagmus, and hypotonia) [14, 15].

*MPV17*, one of the recently discovered genes found to be related to hepatocerebral class, is associated with MDS type 6. It is a ubiquitously expressed gene encoding a highly conserved protein in the inner mitochondrial membrane. MDS type 6 is usually characterized by infantile- or childhood-onset progressive hepatic disease, neurological defects as well as metabolic manifestations such as lactic acidosis and hypoglycemia [16–18]. However, there have been reports of adult-onset mtDNA deletion disease due to *MPV17* mutations [19, 20]. In contrast to other forms of MDS, neurological defects are usually milder on presentation in MDS caused by mutations in *MPV17* [21].

According to some investigations, the levels of tyrosine and phenylalanine are elevated in blood or urine of those with *DGUOK* and *MPV17* mutations found in their newborn screening [22, 23]. Tyrosinemia is an inborn error of metabolism caused by impaired tyrosine metabolism [24]. Herein, we report on the disease-causing mutations in three families affected with hepatocerebral form of MDS who were initially diagnosed with tyrosinemia.

## Case presentation

### Whole-exome sequencing (WES)

WES was carried out on whole blood samples taken from parents of all patients to capture and enrich all exons of protein coding genes in addition to other essential parts of the genome. Next generation sequencing (NGS) was performed using Illumina Hiseq 2000 machine to sequence close to 100 million reads and standard Illumina protocol for pair-end 99 nucleotide sequencing. Basically, the test platform assayed > 95% of the target regions with sensitivity of above 99%.

### Sanger sequencing

To confirm the novel mutations, whole blood samples were collected from healthy parents of all three families in EDTA tubes. All probands of the affected families had died at the time of investigation. However, we had access to the extracted DNA sample from a daughter of family II that was kept in hospital at − 20 °C, dry umbilical cord that belonged to the affected son of family III, and chorionic villus sample (CVS) from the 7-week pregnant mother of family I. DNA was extracted from all samples (blood, CVS, tissue) using QIAamp DNA Minikit (Qiagen, Germany) according to the manufacturer’s instructions. The DNA concentration was then evaluated by Epoch Microplate Spectrophotometer (Bio Tek Instruments, USA) and stored at − 20 °C until use.

Following oligonucleotide PCR primers were utilized to amplify the desired genome regions: *DGUOK* (F-Exon5: 5′ AAGACTGCATTGTAGCAG 3′ and R-Exon5: 5’CAGCAATATTAAACTTCTGAGT 3′)*, MPV17* (F-Exon4: 5′ AGTGAGGTAGAGGCCTAG3’ and R-Exon4: 5′ CTGCACCATAACCCTCAG 3′), *MPV17* (F-Exon7: 5′ TGGTGCAGGAATGTGCTC 3′ and R-Exon7: 5′ CTGCAGCCTAGGTTAGAC 3′).

Sanger sequencing was then conducted in both directions on the amplified DNA segments using ABI BigDye Terminator Cycle Sequencing kit (Applied Biosystems®, USA).

### Real-time PCR

Total RNA was isolated from whole blood sample taken from heterozygous parents and also umbilical cord tissue of the deceased homozygous individual in family III using Invitrogen TRIzol Reagent according to the company protocol. RNA concentration, purity and integrity was then measured by Epoch Microplate Spectrophotometer (Bio Tek Instruments, USA). The resulting RNA samples were used for cDNA synthesis using Fermentas cDNA synthesis kit (Thermo Fisher Scientific, USA). We used a Rotor-Gene Q (QIAGEN, Germany) real-time PCR cycler by Invitrogen SYBER Green Master Mix to evaluate any alteration in *DGUOK* and *MPV17* gene expression of parents’ blood and umbilical cord compared with that of a normal control. The following primer pairs were used to assess *DGUOK* and *MPV17* expression: *DGUOK*-QPCR-F: 5′ TGGGAAAGTCCACGTTTGTGAA 3′, *DGUOK*-QPCR-R: 5′ AATGTGTAGGACCATCGTGCTG 3’and *MPV17*-QPCR-F: 5′ ACTACAGCGGGATTATCCT 3′, *MPV17*-QPCR-R: 5′ TAACAGCAACACATTGGAC 3′. We also used glyceraldehyde 3-phosphate dehydrogenase (*GAPDH*) as the reference gene by these primer pairs: *GAPDH*-QPCR-F: 5′ ACAACTTTGGTATCGTGGAAGG 3′, *GAPDH*-QPCR-R: 5’GCCATCACGCCACAGTTTC3’.

Differences in the relative gene expression was evaluated by cycle threshold (Ct) values. We used 2^-∆∆Ct^ method to calculate relative expression between mentioned genes and *GAPDH* gene as the internal control.

### Bioinformatics

Herein, several bioinformatics analyses were conducted using a wide variety of software programs. BWA aligner was used for aligning sequence reads (obtained from WES) against human genome [25]. Genome variants were identified by GATK [26]; then annotated using ANNOVAR software [27]. Public databases and standard bioinformatics software programs, such as CADD_phred, SIFT, Polyphen, Phastcons, LRT, Mutation Taster, and Mutation Assessor were used to evaluate the NGS results.

I-TASSER server (https://zhanglab.ccmb.med.umich.edu/I-TASSER/) was used to predict 3D structure of proteins [28, 29]. Wild-type and mutant protein structure of proteins were then compared with UCSF chimera.

Multiple sequence alignment software program was also used to conduct comparative amino acid sequence alignment of MPV17 and DGUOK proteins.

### Family Ι: patients Ι

Patient I was a 6-month-old girl, known case of liver cirrhosis, who presented with dyspnea, bloody vomiting, diarrhea, lethargy, jaundice, and dark urine. She was the second child of non-symptomatic parents who were first-degree cousins. The first child of the family presenting with similar symptoms had died at the age of 3 months. Both children had impaired fetal growth, low-birth weight, and failure to thrive. Both were floppy and hypotonic and developed prolonged jaundice and green-colored stool. The first infant weighted 1700 g at birth, had Apgar scores of 8 and 9 at the 1st and 5th min, respectively. The second child weighted 2500 g with respective Apgar scores of 8 and 8. Moreover, the first infant suffered from an unspecified ophthalmologic problem. She died at the age of 3 months due to progressive respiratory insufficiency.

On physical examination, stridor was heard over the entire lung fields on auscultation. The abdomen was distended with mild free ascitic fluid. Her vital signs included a heart rate of 142 beats/min, respiratory rate of 44 breaths/min, axillary temperature of 36.9 °C, and peripheral blood O_2_ saturation of 56% on breathing the ambient air.

Laboratory evaluations indicated increased serum ALT, AST, Alk-P, AFP, and total bilirubin, and prolonged PT and PTT (Table 1). Viral markers were negative. Immunoglobulin levels were within normal range. Blood sample assay showed slightly decreased biotinidase and normal Gal-P urodyltransferase activities. Tandem mass spectrometry showed elevated level of phenylalanine, tyrosine, and methionine. Based on clinical picture, the patient was initially diagnosed as having either tyrosinemia or galactosemia. On the third day of admission, the patient died. The family was referred for genetic counselling to find out whether the underlying problem was hereditary and to terminate the current pregnancy if necessary.

**Table 1:** Laboratory findings in the patients showing increased Alk-P, AFP, and blood tyrosine levels in all affected individuals

Variable	Patient I	Patient II	Patient III
AST (Reference range: < 60 U/L)	942	241	176
ALT (Reference range: < 45 U/L)	554	106	61
Alk-P (Reference range: 124–341 U/L)	1274	897	2700
AFP (Reference range: 0–97 ng/mL)	16,372	291	> 2000
Total Bilirubin (Reference range: < 1.9 mg/dL)	12.8	11.9	10.5
Direct Bilirubin (Reference range: < 0.2 mg/dL)	3.2	4.7	3.9
PT (Reference range: 10.5–11.5 s)	> 60	21.8	34
PTT (Reference range: 24–36 s)	58	59	58
Blood tyrosine (Reference range: 20–100 μmol/L)	240	176	309
Blood succinylacetone (Reference range: < 5.0 mcM)	< 5.0	< 5.0	< 5.0

**Fig. 1:**
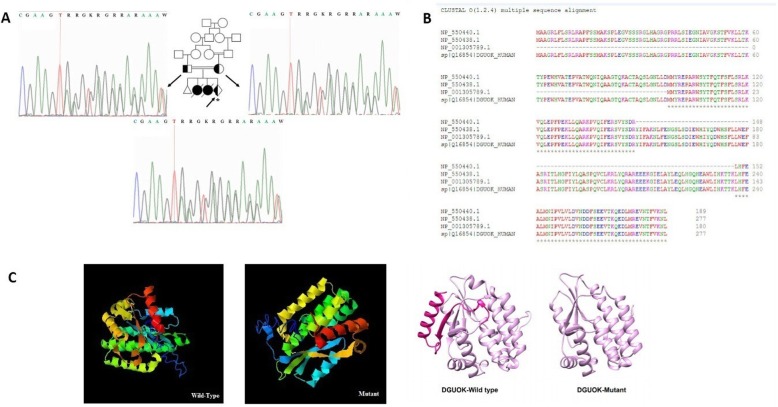
**a** Family I pedigree and sequence chromatograms. Both parents are heterozygous for the frameshift deletion mutation in *DGUOK* gene. The current pregnancy was a heterozygous carrier of the mutation. The proband is marked by an asterisk. **b** Clustal Omega multiple sequence alignment of all functional human encoding isoforms of DGUOK protein showing that all functional isoforms share the same amino acid sequence after codon 236. **c** Three dimensional structure of DGUOK wild type and mutant proteins were predicted by I-TASSER server. UCSF chimera was used to compare the structures. The deleted region is displayed in pink

NGS data analysis revealed a pathogenic heterozygous frameshift deletion mutation in *DGUOK* gene (*DGUOK*: NM_001318860:exon5:c.706_707 + 2del:p.K236 fs) that was present in both parents. Homozygous or compound heterozygous *DGUOK* gene mutations have been reported in hepatocerebral MDS. Sanger sequencing confirmed the heterozygous frameshift deletion in the parents. Therefore, the disease might be inherited as an autosomal-recessive trait. Sanger sequencing study of the CVS sample revealed that the current pregnancy was a heterozygous carrier of the mutation (Fig. 1a).

**Fig. 2:**
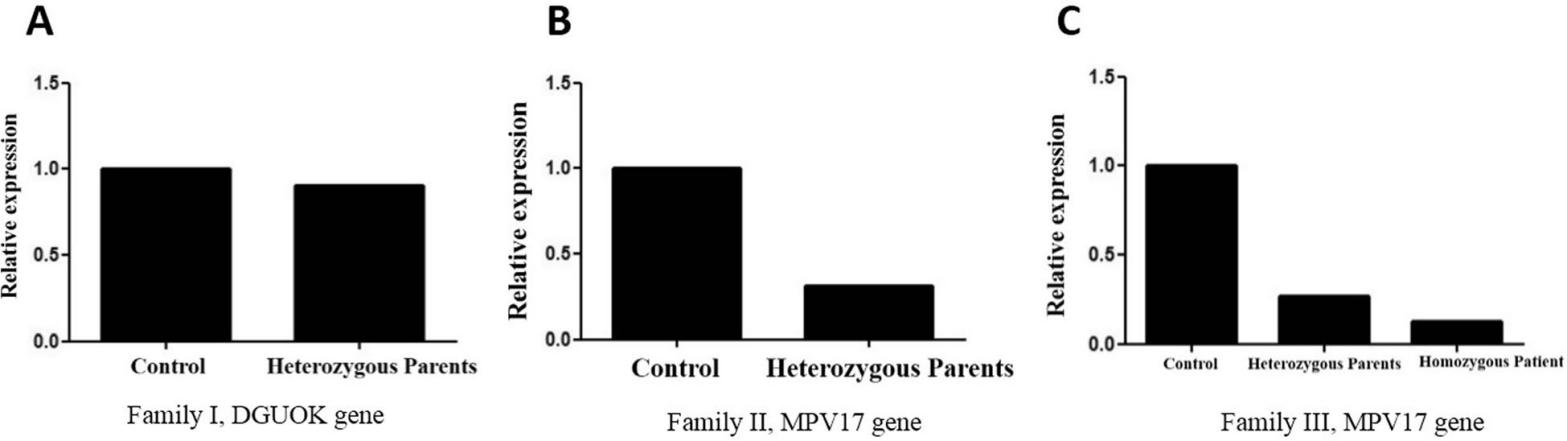
Quantitative real-time PCR results. **a** *DGUOK* mRNA expression levels show no significant differences between heterozygous parents and normal individual. **b** In family II, *MPV17* mRNA expression reduced in heterozygous parents compared with normal control. **c** Real-time PCR analysis of *MPV17* gene in parents and the affected proband in family III and normal control, showing significant reduction in mRNA expression level in the proband compared with normal control

Clustal Omega multiple sequencing alignment was used to align different functional isoforms of *DGUOK* proteins. The result showed that all functional isoforms of *DGUOK* protein shared the same amino acid sequence after the position 236 (Fig. 1b). This highlighted the vital role of these amino acids in the protein’s functions. As mentioned above, the exact position of the observed deletion mutation was at amino acid 236 (p.K236 fs), which could be an evidence for pathogenicity of this mutation. Mutation taster online software was also used and predicted this variation as a disease causing variant resulting in a non-sense mediated decay at position 239 in the mutant amino acid sequence [30]. 3D structure of wild-type and mutant proteins were predicted using I-TASSER server [28, 29]. UCSF chimera was used to compare these two structures (Fig. 1c). Real-time PCR analysis of DGUOK mRNA expression level revealed no significant difference between parents (heterozygous carriers) and healthy control (Fig. 2a).

### Family ΙΙ: patient ΙΙ

The second patient was a 4-month-old girl who presented with jaundice since one month prior to admission. She also had weak crying, muscle weakness, poor sucking, and failure to thrive. Being a product of full-term normal vaginal delivery, she had normal APGAR score, and birth weight and head circumference. The patient had an episode of seizure when she was 12 days old. The parents were second-degree cousins and had a younger sibling who had died at the age of 9 months due to an unknown metabolic disorder. The mother also reported a previous abortion.

Detailed neurological examination revealed neurodevelopmental delay and muscle weakness in patient II. The “Fix and Follow test” of moving objects was abnormal. The physical examinations were otherwise unremarkable. She had no abnormal findings on brain MRI. Diagnostic laboratory evaluations revealed elevated serum AST, ALT, AFP, and prolonged PT and PTT (Table 1). Tyrosine level was also elevated. Liver biopsy was in favor of cirrhosis. This patient was also initially diagnosed as having tyrosinemia. She died of hepatic failure at the age of 4 months.

**Fig. 3:**
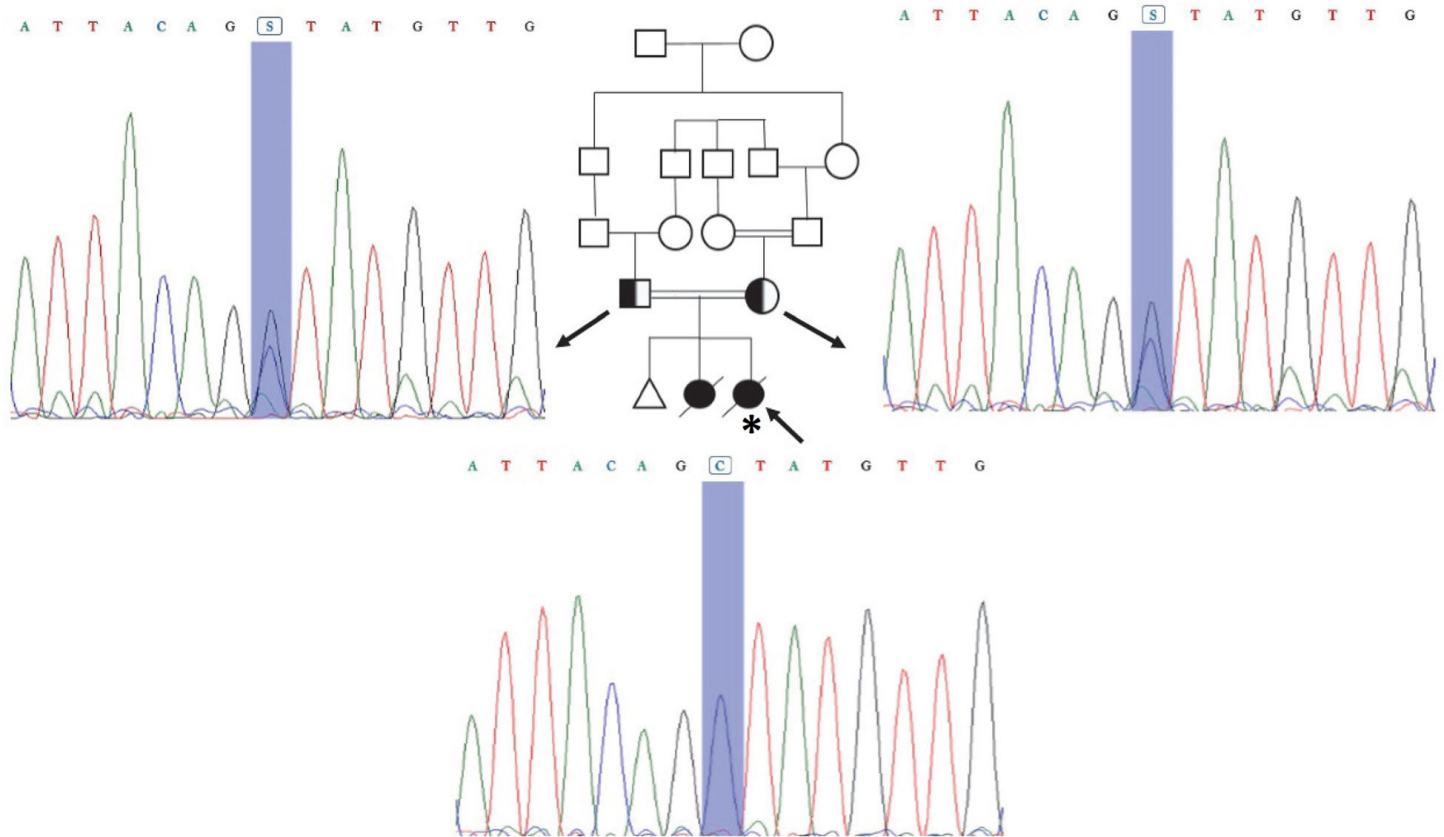
Family II pedigree and sequence chromatograms. Both parents are heterozygous and the affected individual is homozygous for this mutation in *MPV17* gene. The proband is marked by an asterisk

NGS results showed a novel heterozygous missense (splice donor site) mutation in *MPV17* gene (*MPV17*: NM_002437:exon7:c.461 + 1G > C) in the parents. Mutation in this gene can cause autosomal-recessive mitochondrial DNA depletion syndrome 6 (hepatocerebral type). Sanger sequencing confirmed heterozygosity and homozygosity for the mentioned splice site donor mutation in both parents and the patient, respectively, indicating the autosomal-recessive inheritance pattern for this disease (Fig. 3). According to the mutation taster, this variant is disease causing leading to change in the conserved splice site nucleotide. Conservation analysis of this nucleotide also revealed a Phylop score of 3.966 and a Phastcons score of 1.

Analysis of *MPV17* mRNA using real-time PCR on heterozygous parents clearly indicated significant decreased level of *MPV17* mRNA expression compared with normal control. It can be concluded that one mutated copy of *MPV17* gene can affect the expression of this gene (Fig. 2b). However, one copy of this gene is sufficient to provide functional MPV17 protein in heterozygous carrier*.* Since we did not have RNA from the deceased child, real-time PCR only was performed on a heterozygous carrier. As a result, the mutation in *MPV17* gene described here can contribute to hepatocerebral MDS.

### Family ΙΙΙ: patient ΙII

A 5-month-old boy, whose parents were first-degree cousins, was brought to the Pediatric Neurology ward of Namazi Hospital due to malaise, jaundice, dark urine, poor sucking, and feeding problems. He also had neurodevelopmental delay. The patient was the second child of a healthy couple and was a product of full-term normal vaginal delivery. He had normal APGAR scores. His weight and head circumference were appropriate at birth and during infancy.

He had previous history of hospital admission at the age of 16 days for prolonged jaundice, dark urine, and lethargy. Moreover, his serum ferritin level, AFP, AST, ALT, Alk-P, and total bilirubin levels were high (Table 1). All viral markers studied were negative. Ophthalmologic and gastrointestinal studies did not show any abnormalities. He also had an abnormal electroencephalogram (EEG) with epileptiform activities. Due to high serum phenylalanine and tyrosine levels in PKU screening (using tandem mass spectrometry), the patient was initially treated with PKU formula and phenobarbital. He was subsequently discharged with close clinical follow-up. He was apparently well until the age of 5 months when he was admitted with the primary diagnosis of tyrosinemia due to high tyrosine level in his serum. Otherwise, the physical examinations showed no abnormalities. Laboratory evaluations revealed increased serum AST, ALT, Alk-P, AFP, total and direct bilirubin, and tyrosine levels, and prolonged PT and PTT (Table 1). The patient died on the second day of admission.

**Fig. 4:**
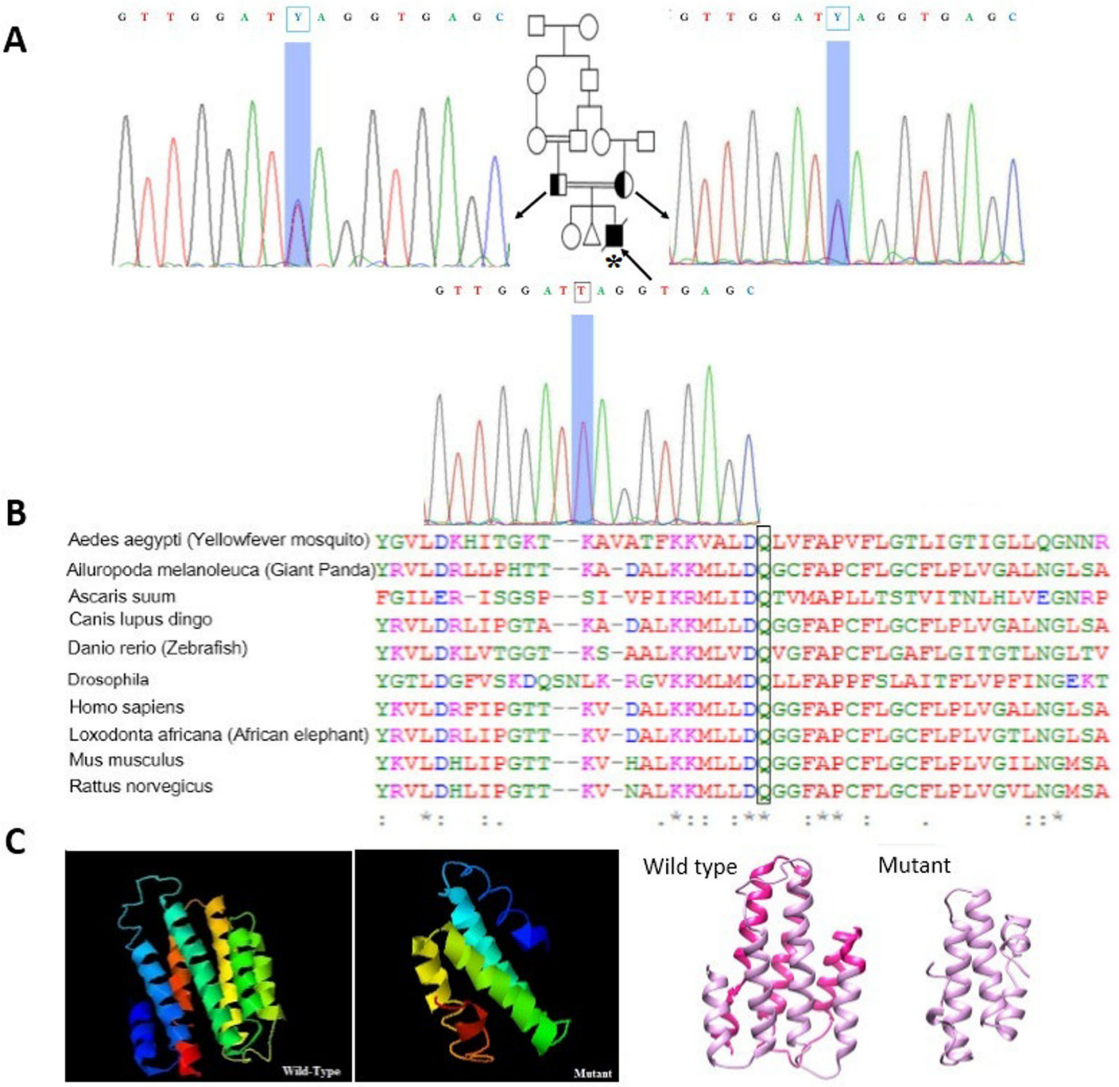
**a** Family III pedigree and sequence chromatograms. Both parents are heterozygous and the affected proband is homozygous for this stop-gain mutation in *MPV17* gene. The proband is marked by an asterisk. **b** Comparative amino acid alignment of MPV17 protein across different kingdoms. The conserved glutamine residue is shown in the box. **c** Protein structure was predicted by I-TASSER server for 3D protein structure prediction. The secondary structure of protein is changed as a result of the mutation. The deleted region is showed in pink

NGS analysis results on parents revealed a novel heterozygous nonsense (stop gain) mutation in another region of *MPV17* gene (*MPV17:* NM_002437: c.277C > T: p.(Gln93*)). Sanger sequencing revealed that both parents were heterozygous and the proband was homozygous for this mutation (Fig. 4a). Mutation taster predicted that this variation is a disease causing variant. The comparative amino acid alignment of *MPV17* protein was conducted across different animal kingdoms using multiple sequence alignment analysis by T-Coffee Multiple Sequence Alignment Program. The Q93 residue was highly conserved (Fig. 4b). In addition, prediction of the 3D protein structure using I-TASSER server [28, 29], revealed structural alterations resulting from this stop-gain mutation (Fig. 4c).

Real-time PCR was performed on three members of this family. mRNA expression in normal individuals was significantly higher than that in parents and the proband. It clearly demonstrated the depletion in MPV17 expression in the proband (Fig. 2c).

## Discussion and conclusions

MDS is a heterogeneous disorder that results from a reduction in mtDNA copy number. It can lead to a wide range of clinical presentations due to insufficient synthesis of the respiratory chain complexes (I, III, IV, V), which ultimately leads to insufficient energy production and mitochondrial dysfunction [6, 7, 31].

Hepatocerebral type of MDS often occurs in the infancy and its common early symptoms include persistent vomiting, failure to thrive, hypotonia, and hypoglycemia. To date, numerous genes associated with MDS have been identified. Mutations in *DGUOK* and *MPV17*, which are involved in mtDNA maintenance, have been reported in the hepatocerebral form of the disorder [2]. In this paper, we report on three hepatocerebral MDS cases which resulted from mutations of *DGUOK* gene in one family and *MPV17* gene in two families.

*DGUOK* has seven exons, encoding the mitochondrial deoxyguanosine kinase, which supplies dNTP for mtDNA replication. Mandel, *et. al.*, illustrated a region on chromosome 2p13 by homozygosity mapping that included *DGUOK* gene in three families with hepatocerebral MDS. It was found that a nucleotide deletion (204 del A) in *DGUOK* segregated with the disease [31, 32].

With advancements in genetics sequencing techniques, especially next generation sequencing, detecting these mutations has become easier. According to The Human Gene Mutation Database (HGMD), 57 mutations have so far been reported in *DGUOK* gene—34 missense/nonsense, 6 splicing, 9 small deletions, 5 small insertions and 3 gross deletions. These mutations affect both the conserved and non-conserved *DGUOK* amino acids [33].

Whole exome sequencing of the couple in family I illustrated a deleterious heterozygous frameshift deletion mutation in *DGUOK* gene in both parents. Frameshift mutations may indirectly cause a premature termination codon and give rise to translation reading frameshift or sometimes altered splicing [34].

The parents of the patient in family I were carriers of a mutation resulting in deletion of four nucleotides (c.706–707 + 2 del AAGT) located in a splice donor site. Sezer, *et. al*., reported a 2-month-old girl with deletion mutation of DGUOK gene in this region (c.707 + 3–6 del TAAG) that affects splicing site and causes mitochondrial DNA depletion syndrome [35]. Here we report another patient with a deleterious mutation in this region.

In this study, the heterozygous parents showed normal DGUOK mRNA expression compared to normal control, reflecting that one mutated copy of this gene had no effect on mRNA expression. However, in silico investigations revealed that AAGT deletion could ultimately result in premature translation termination after codon 236, producing a truncated protein and might therefore affect the 3D protein structure or lead to non-sense mediated decay at position 239 in the mutant amino acid sequence; the wild type stop codon is located at position 278 in the amino acid sequence.

Three-dimensional protein structure of DGUOK was identified in 2001; two domains (β-5 and α-9) were discovered after position 245 [36]. Wang, *et. al.*, showed that the C-terminal α-helix number 9 (α-9) domain of DGUOK has a vital role in enzyme activity as part of phosphate donor binding site [37]. Therefore, it seems that the identified frameshift deletion in *DGUOK* gene could result in DGUOK deficiency and cause MDS type 3 syndrome in family I.

Patients with *DGUOK* deficiency would eventually show early-onset liver failure, which is the most common symptom. However, neurological involvement may be mild or absent [38]. Our patient had hepatomegaly and failure to thrive and died of liver failure. It is worth mentioning that the levels of three amino acids—phenylalanine, tyrosine, and methionine—were elevated in tandem mass spectrometry results of newborn screening. The observation is associated with liver failure and can be observed in large number of newborns affected by multiorgan form of the disease. Elevated hepatic enzymes concentration in the serum, direct hyperbilirubinemia, and increased gamma-glutamyltransferase levels were also observed in the affected infants, reflecting intrahepatic cholestasis [39].

Spinazzola, *et. al.*, reported a new locus for hepatocerebral MDS on chromosome 2p21–23 by genome wide linkage analysis. *MPV17* was one of the top candidate genes. *MPV17* mutations segregated with the disease in all affected families [17]. The gene has eight exons, encoding a mitochondrial inner membrane protein. Although its main function is still unknown, loss of function in this protein has been shown to cause aberrant oxidative phosphorylation (OXPHOS) and mtDNA depletion in *MPV17* knock out mice model [40].

*MPV17* protein, which was assumed to be located in peroxisome, has been proven to be localized in the mitochondria [17]. So far, 37 pathogenic variants have been reported in *MPV17* genes on The Human Gene Mutation Database (HGMD)—19 missense/nonsense mutations, 5 splicing variants, 6 small deletions, 2 small insertions, 1 small indels and 4 gross deletions. Most of these mutations were reported as private, except p.Arg50Gln mutation, which is the most common form [41].

In the current study, a novel missense mutation was identified in family II at a conserved splice donor site (GT at the 5′ end of an intron). It causes inaccurate pattern of RNA splicing that leads to exon omission (exon skipping) or failure to splice out an intron (intron retention). Abnormal splicing may lead to a frameshift mutation at the RNA level, which induces RNA degradation or production of a truncated protein [30]. As real-time PCR results suggested, *MPV17* was significantly down-regulated in the heterozygous parents. This could be attributed to asymmetrical degradation of different splicing forms resulting from the mutation or the detrimental effect of the pathogenic variant on the regions of the gene most important for its expression.

In family III, the results showed a novel nonsense mutation (stop gain) in exon 4 of *MPV17*, which changed the p.93Q into the stop codon. The resultant mRNA might be targeted by a mechanism known as nonsense-mediated decay. The Q93 residue is highly conserved during evolution, which conveys the crucial role of glutamine in this residue. Position of the stop codon in the wild type amino acid sequence is in codon 177, while that of the mutant one is in codon 93. Therefore, the mutation would significantly affect the protein structure, which was also shown using I-TASSER server for 3D protein structure prediction. According to the Pfam database, there is a potential transmembrane helical structure might exist from codon 95 to 115 of the protein and an early stop codon in position 93 can thus disrupt the protein structure. Analysis of mRNA expression results showed that *MPV17* was significantly down-regulated in the proband compared with a normal homozygous individual. On account of the evidence presented, it can be concluded that this mutation can give rise to hepatocerebral MDS disorder.

No successful treatment has so far been found for patients with mitochondrial hepatopathy. Researchers have conducted investigations into the effect of various vitamins, cofactors and respiratory substrates for the treatment of these patients. However, none of these interventions have been found effective. Liver transplantation can only be partly effective in the absence of neurological symptoms [9].

We found one mutation in *DGUOK* gene and two novel mutations in *MPV17* in patients affected by hepatocerebral MDS. Due to increased serum tyrosine levels in most of the affected individuals, MDS has overlapping clinical features with tyrosinemia. Therefore, suspected cases should be provided with professional genetic counselling to give correct diagnosis. Moreover, physicians should be more aware of MDS as a differential diagnosis in patients with such overlapping symptoms. Molecular diagnosis may be designed to help in identifying MDS patients and families and establish accurate prenatal diagnosis of high risk individuals.

## Supplementary information

**Additional file 1: Table S1.** Different genes associated with mitochondrial DNA depletion syndrome.

## Data Availability

All data are available from the corresponding author on request.

## Abbreviations

AFP: Alpha Fetoprotein
Alk-P: Alkaline phosphatase
ALT: Alanine transaminase
AST: Aspartate transaminase
CVS: Chorionic villus sampling
EEG: Electroencephalogram
MDS: Mitochondrial DNA depletion syndromes
mtDNA: Mitochondrial DNA
NGS: Next generation sequencing
PKU: Phenylketonuria
PT: Prothrombin time
PTT: Partial thromboplastin time
WES: Whole-Exome Sequencing
