# Supplementary material for: Clinical and molecular characterization of three patients with Hepatocerebral form of mitochondrial DNA depletion syndrome: a case series
Source: BMC Med Genet. 2019 Oct 29;20:167. doi: 10.1186/s12881-019-0893-9 (PMC6819644; doi:10.1186/s12881-019-0893-9)
Supplement: Supplementary file 1 — Additional file 1: Table S1. Different genes associated with mitochondrial DNA depletion syndrome. [file 12881_2019_893_MOESM1_ESM.docx]

| Table S1 | | | | |
| --- | --- | --- | --- | --- |
| Reference | Phenotype MIM number | Clinical features | Gene/Locus MIM number | Gene Symbol |
| [1] | 603041 | MTDPS1 | 131222 | *TYMP* |
| [2] | 609560 | MTDPS2 | 188250 | *TK* |
| [3] | 251880 | MTDPS3 | 601465 | *DGUOK* |
| [4, 5] | 203700,613662 | MTDPS4 A,B | 174763 | *POLG1* |
| [6] | 612073 | MTDPS5 | 603921 | *SUCLA2* |
| [7] | 256810 | MTDPS6 | 137960 | *MPV17* |
| [8] | 271245 | MTDPS7 | 606075 | *TWNK* |
| [9, 10] | 612075 | MTDPS8 A,B | 604712 | *RRM2B* |
| [11] | 245400 | MTDPS9 | 611224 | *SUCLG1* |
| [12] | 212350 | MTDPS10 | 610345 | *AGK* |
| [13] | 615084 | MTDPS11 | 615076 | *MGME1* |
| [14, 15] | 617184,615418 | MTDPS12 A,B | 103220 | *SLC25A4* |
| [16] | 615471 | MTDPS13 | 605654 | *FBXL4* |
| [17] | 616896 | MTDPS14 | 605290 | *OPA 1* |
| [18] | 617156 | MTDPS15 | 600438 | *TFAM* |

1. Nishino I, Spinazzola A, Hirano M: **Thymidine phosphorylase gene mutations in MNGIE, a human mitochondrial disorder**. *Science* 1999, **283**(5402):689-692.

2. Saada A, Shaag A, Mandel H, Nevo Y, Eriksson S, Elpeleg O: **Mutant mitochondrial thymidine kinase in mitochondrial DNA depletion myopathy**. *Nature genetics* 2001, **29**(3):342.

3. Mandel H, Szargel R, Labay V, Elpeleg O, Saada A, Shalata A, Anbinder Y, Berkowitz D, Hartman C, Barak M: **The deoxyguanosine kinase gene is mutated in individuals with depleted hepatocerebral mitochondrial DNA**. *Nature genetics* 2001, **29**(3):337.

4. Naviaux RK, Nguyen KV: **POLG mutations associated with Alpers' syndrome and mitochondrial DNA depletion**. *Annals of neurology* 2004, **55**(5):706-712.

5. Vissing J, Ravn K, Danielsen E, Dunø M, Wibrand F, Wevers R, Schwartz M: **Multiple mtDNA deletions with features of MNGIE**. *Neurology* 2002, **59**(6):926-929.

6. Jaberi E, Chitsazian F, Shahidi GA, Rohani M, Sina F, Safari I, Nejad MM, Houshmand M, Klotzle B, Elahi E: **The novel mutation p. Asp251Asn in the β-subunit of succinate-CoA ligase causes encephalomyopathy and elevated succinylcarnitine**. *Journal of human genetics* 2013, **58**(8):526.

7. Spinazzola A, Viscomi C, Fernandez-Vizarra E, Carrara F, D'Adamo P, Calvo S, Marsano RM, Donnini C, Weiher H, Strisciuglio P: **MPV17 encodes an inner mitochondrial membrane protein and is mutated in infantile hepatic mitochondrial DNA depletion**. *Nature genetics* 2006, **38**(5):570.

8. Nikali K, Suomalainen A, Saharinen J, Kuokkanen M, Spelbrink JN, Lönnqvist T, Peltonen L: **Infantile onset spinocerebellar ataxia is caused by recessive mutations in mitochondrial proteins Twinkle and Twinky**. *Human molecular genetics* 2005, **14**(20):2981-2990.

9. Bourdon A, Minai L, Serre V, Jais J-P, Sarzi E, Aubert S, Chrétien D, de Lonlay P, Paquis-Flucklinger V, Arakawa H: **Mutation of RRM2B, encoding p53-controlled ribonucleotide reductase (p53R2), causes severe mitochondrial DNA depletion**. *Nature genetics* 2007, **39**(6):776.

10. Shaibani A, Shchelochkov OA, Zhang S, Katsonis P, Lichtarge O, Wong L-J, Shinawi M: **Mitochondrial neurogastrointestinal encephalopathy due to mutations in RRM2B**. *Archives of neurology* 2009, **66**(8):1028-1032.

11. Ostergaard E, Christensen E, Kristensen E, Mogensen B, Duno M, Shoubridge EA, Wibrand F: **Deficiency of the α subunit of succinate–coenzyme A ligase causes fatal infantile lactic acidosis with mitochondrial DNA depletion**. *The American Journal of Human Genetics* 2007, **81**(2):383-387.

12. Calvo SE, Compton AG, Hershman SG, Lim SC, Lieber DS, Tucker EJ, Laskowski A, Garone C, Liu S, Jaffe DB: **Molecular diagnosis of infantile mitochondrial disease with targeted next-generation sequencing**. *Science translational medicine* 2012, **4**(118):118ra110-118ra110.

13. Kornblum C, Nicholls TJ, Haack TB, Schöler S, Peeva V, Danhauser K, Hallmann K, Zsurka G, Rorbach J, Iuso A: **Loss-of-function mutations in MGME1 impair mtDNA replication and cause multisystemic mitochondrial disease**. *Nature genetics* 2013, **45**(2):214.

14. Thompson K, Majd H, Dallabona C, Reinson K, King MS, Alston CL, He L, Lodi T, Jones SA, Fattal-Valevski A: **Recurrent de novo dominant mutations in SLC25A4 cause severe early-onset mitochondrial disease and loss of mitochondrial DNA copy number**. *The American Journal of Human Genetics* 2016, **99**(4):860-876.

15. Palmieri L, Alberio S, Pisano I, Lodi T, Meznaric-Petrusa M, Zidar J, Santoro A, Scarcia P, Fontanesi F, Lamantea E: **Complete loss-of-function of the heart/muscle-specific adenine nucleotide translocator is associated with mitochondrial myopathy and cardiomyopathy**. *Human molecular genetics* 2005, **14**(20):3079-3088.

16. Bonnen PE, Yarham JW, Besse A, Wu P, Faqeih EA, Al-Asmari AM, Saleh MA, Eyaid W, Hadeel A, He L: **Mutations in FBXL4 cause mitochondrial encephalopathy and a disorder of mitochondrial DNA maintenance**. *The American Journal of Human Genetics* 2013, **93**(3):471-481.

17. Spiegel R, Saada A, Flannery PJ, Burté F, Soiferman D, Khayat M, Eisner V, Vladovski E, Taylor RW, Bindoff LA: **Fatal infantile mitochondrial encephalomyopathy, hypertrophic cardiomyopathy and optic atrophy associated with a homozygous OPA1 mutation**. *Journal of medical genetics* 2016, **53**(2):127-131.

18. Stiles AR, Simon MT, Stover A, Eftekharian S, Khanlou N, Wang HL, Magaki S, Lee H, Partynski K, Dorrani N: **Mutations in TFAM, encoding mitochondrial transcription factor A, cause neonatal liver failure associated with mtDNA depletion**. *Molecular genetics and metabolism* 2016, **119**(1):91-99.
